# Process of building an entrepreneurial career in Nursing

**DOI:** 10.1590/1980-220X-REEUSP-2023-0086en

**Published:** 2023-11-20

**Authors:** Vinícius Luís da Silva, Dandara Novakowski Spigolon, Hellen Emília Peruzzo, Maria Antonia Ramos Costa, Verusca Soares de Souza, Heloá Costa Borim Christinelli, Edilaine Maran, Maria Luiza Costa Borim

**Affiliations:** 1 Universidade Estadual do Paraná, Paranavaí, PR, Brazil.; 2 Universidade Federal de Mato Grosso do Sul, Coxim, MS, Brazil.; 3 Universidade Estadual de Maringá, Maringá, PR, Brazil.

**Keywords:** Entrepreneurship, Nursing, Health Sciences, Technology, and Innovation Management, Creativity, Nursing Administration Research, Emprendimiento, Enfermería, Gestión de Ciencia, Tecnología e Innovación en Salud, Creatividad, Investigación en Administración de Enfermería, Empreendedorismo, Enfermagem, Gestão de Ciência, Tecnologia e Inovação em Saúde, Criatividade, Pesquisa em Administração de Enfermagem

## Abstract

**Objective:**

To learn about the experiences of nurse entrepreneurs in building their careers and business trajectories.

**Method:**

A descriptive and exploratory, qualitative study carried out with nurse entrepreneurs, recruited using the snowball technique. Interviews were conducted via Skype and audio-recorded between June and July 2021, which were transcribed and subjected to content analysis.

**Results:**

15 nurses participated. Three thematic axes were identified: “Entrepreneurship in nursing with the use of technologies”, which presented niches of activity and the use of technologies; “Desire to innovate even with fear of the new”, which reports feelings and motivations related to entrepreneurship, especially innovation; and “What nurses need to know before starting a business”, which presents the risks and benefits of entrepreneurship, as well as the skills needed by entrepreneurs.

**Conclusion:**

The entrepreneurial experience presents challenges for nurses. Thus, encouraging knowledge about entrepreneurship makes it possible to strengthen autonomy and gain new opportunities in nursing.

## RESUMO

**Objetivo:**

Conhecer as experiências de enfermeiros empreendedores na construção da carreira e trajetória empresarial.

**Método:**

Estudo descritivo e exploratório, qualitativo, realizado com enfermeiros empreendedores, recrutados pela técnica “Bola de Neve”. Realizou-se entrevistas via Skype e audiogravadas nos meses de junho a julho de 2021, que foram transcritas e submetidas a análise de conteúdo.

**Resultados:**

Participaram 15 enfermeiros. Identificou-se três eixos temáticos: “Empreender na enfermagem com a utilização de tecnologias”, que apresentou nichos de atuação e o uso de tecnologias; “Desejo de inovar mesmo com medo do novo”, que relata sentimentos e motivações relacionados ao empreender, em especial a inovação; e “O que o enfermeiro precisa saber antes de empreender”, que apresenta os riscos e benefícios do empreendimento, bem como, as habilidades necessárias ao empreendedor.

**Conclusão:**

A experiência empreendedora apresenta desafios na prática dos enfermeiros. Destarte, incentivar o saber sobre o empreendedorismo possibilita o fortalecimento da autonomia e conquista de novas oportunidades de atuação na Enfermagem.

## RESUMEN

**Objetivo:**

Conocer las experiencias de enfermeras emprendedoras en la construcción de sus carreras y trayectorias empresariales.

**Método:**

Estudio cualitativo descriptivo y exploratorio realizado con enfermeras emprendedoras reclutadas mediante la técnica “Bola de Nieve”. Las entrevistas se realizaron a través de Skype y se grabaron en audio entre junio y julio de 2021, se transcribieron y se sometieron a un análisis de contenido.

**Resultados:**

Participaron 15 enfermeras. Se identificaron tres ejes temáticos: “Emprendimiento en enfermería con el uso de tecnologías”, que presenta nichos de actividad y el uso de tecnologías; “Deseo de innovar aún con miedo a lo nuevo”, que reporta sentimientos y motivaciones relacionados con el emprendimiento, especialmente la innovación; y “Lo que las enfermeras necesitan saber antes de emprender”, que presenta los riesgos y beneficios del emprendimiento, así como las habilidades que necesitan las emprendedoras.

**Conclusión:**

La experiencia emprendedora presenta retos en la práctica de las enfermeras. Por lo tanto, fomentar el conocimiento sobre el espíritu emprendedor permite fortalecer la autonomía y obtener nuevas oportunidades en la enfermería.

## INTRODUCTION

Entrepreneurship has been present in the nursing profession since the 19^th^ century, through Florence Nightingale, who cared for wounded soldiers during the Crimean War, promoting environmental management with the use of an oil lamp for lighting. The intervention in these conditions reduced the proliferation of diseases, having an impact on the recovery of individuals. This practice is considered to be entrepreneurial because of the transformation of a reality that systematizes care^(1)^.

The term “entrepreneurship” means planning, organizing, managing and taking risks in a business or enterprise, with a view to achieving success and growth in the job market^(2)^. In this sense, entrepreneurship can be defined as the act of doing something different, based on identifying unmet needs and proposing innovative and creative solutions in order to patent or register new products and/or services. In addition, implementing health technologies can influence the improvement of nurses’ working environment and contribute to effective care^(3)^.

Entrepreneurial practices in nursing represent a wide and still little-understood field in which nurses can act in various sectors that provide care to prevent disease, promote or recover the health of the population, providing care in various niches, such as offices, homes, cooperatives, consultancies, audits, events, teaching, providing specialized services, among others^(2)^.

Nurses are still “invisible” in the field of entrepreneurship, as many people associate them with health care practices in the context of primary care and hospitals. As a result, entrepreneurial initiatives in the health area in Brazil face some negative determinants and conditioning factors, such as the hospital care model, the medical-centered culture, as well as the lack of knowledge of legislation and the complexity of bureaucratic processes for registering, licensing and managing private businesses. However, despite the advances and entrepreneurial practices that have already been achieved, new possibilities can and should still be developed^(4)^.

In order to overcome the challenges of business management, new perspectives in the 21^st^ century related to the management of science, technology and innovation generate opportunities to work in different roles in the job market. Those new roles, such as the increase in the number of specialties and the support of technology for patient care, which have been explored for entrepreneurship in Nursing with differentiated models in the various areas of care provided to the individual, family and community^(5)^. However, there are still few studies exploring this topic in Nursing, which is a limiting factor in inspiring more professionals to follow this entrepreneurial career path.

Disseminating and demystifying the topic of entrepreneurship in nursing is a technical-scientific contribution that aims to encourage nurses to (re)create and search for innovation in the processes that involve health work, encouraging research into nursing administration, as well as the development and implementation of technologies and innovation in health based on social and institutional needs^(3)^. Given the justifications presented, this study aims to clarify the following question: “How have nurses experienced their careers and entrepreneurial trajectories in nursing?”. In order to answer the research question, the aim of this study was to find out about the experiences of nurse entrepreneurs in building their careers and entrepreneurial careers.

## METHODS

### Study Design

This is a descriptive and exploratory study applying a qualitative approach, which used the Consolidated Criteria for Reporting Qualitative Research (COREQ)^(6)^ checklist for the planning, execution and preparation of the research report. Exploratory research provides greater familiarity with the problem, aimed to making it more explicit or building hypotheses. Its planning tends to be quite flexible, as it is interested in considering the most varied aspects relating to the fact or phenomenon being studied^(7)^.

### Setting and Data Collection Period

The study was carried out via video calls through the Skype^®^ platform due to the social distancing imposed by the Covid-19 pandemic, during the months of June to July 2021.

### Study Sampling

The studied population encompassed fifteen (15) nursing graduates. It corresponds to a non-probabilistic sample because entrepreneurship in nursing is a field in growing development, still being explored, and because the qualitative method was established in conducting this study. It should be noted that sampling was carried out using the snowball technique^(8)^.

### Selection Criteria

The inclusion criteria for taking part in the study were being a nurse entrepreneur and working with any type of health technology (soft, soft-hard and hard), nationwide, and belonging to any state in the Brazilian Federation. Nurses who were entrepreneurs in areas not covered by nursing were excluded.

### Recruitment of Participants

For data collection, the snowball technique was applied, known as snowball sampling, in which the individuals selected to be studied invite new participants from their network of friends and acquaintances with the desired characteristics, with the aim of continuing data collection with the target audience until the sampling frame becomes saturated, i.e. there are no new names offered or the names found do not bring new information to the analysis frame^(8)^.

**Figure 1:**
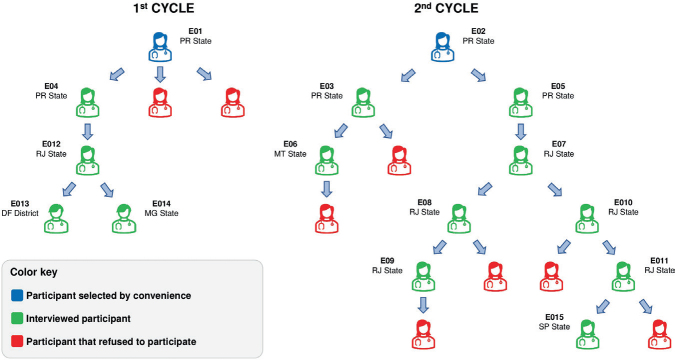
Exponential non-probabilistic sampling using the snowball technique.

The first two participants from the state of Paraná were recruited for convenience, in order to meet the initial needs and facilitate access to the sample, through the WhatsApp application and they consequently provided contacts of other professionals to continue the data collection, as shown in Figure 1.

The study conducted individual, audio-recorded interviews on the Skype^®^ platform, lasting an average of 55 minutes. The information was collected using a semi-structured questionnaire designed by the researchers to determine the sociodemographic and educational characteristics of the participants, in order to investigate how entrepreneurial practices are carried out in their work environment.

The study’s guiding question was: “Could you describe in a few words what your experience has been like during your entrepreneurial career in nursing?”. In addition to the guiding question, four supporting questions were used, when necessary, to further engage the participants during the interview and elucidate the “entrepreneurial career in nursing” phenomenon: What are the challenges of entrepreneurship for nursing?; What was your motivation to become an entrepreneur in nursing?; What are the advantages and disadvantages of being self-employed?; and, In your opinion, what characteristics and skills do nurse entrepreneurs need to expand their business?

### Data Analysis and Processing

All the interviews were transcribed in full. The data was analyzed and processed using the thematic content analysis proposed by Bardin^(9)^. The stages of pre-analysis, exploration of the material and inference of the results were followed. In the pre-analysis stage, exhaustive readings were made of the study participants’ reports. Secondly, in the material exploration stage, the raw data was coded and transformed into meaningful information. The process was then carried out by means of thematic analysis, generating nuclei of meaning, which were brought together by approximating their meanings and resulted in categories. Finally, in a third step, based on the speeches, the inference variables were used to draw up the interpretations. It should be noted that the excerpts/extracts/fragments presented in the results have undergone spelling corrections, without altering the meaning of the speeches, and jargon and repetition have been removed.

### Ethical Aspects

The study followed all the ethical precepts of Resolution no. 466/2012 and Resolution no. 580/2018 of the National Health Council, was approved by the Research Ethics Committee of the State University of Paraná under number 4.781.393 on June 15, 2021. Participants were coded with the acronym “E” for nurse followed by “0” and the Arabic number corresponding to the order of the interview in order to preserve anonymity. For example: “E01”; “E015”.

## RESULTS

### Characterization of the Participants

The study included fifteen specialist nurses working in different areas of nursing. They were predominantly women (86.7%), married (40.0%), aged between 21 and 40 (66.7%), with up to 12 years of training (46.7%), and who were trained in entrepreneurship (73.3%).

Regarding the characterization of the nurse professionals’ ventures, there was a predominance of micro-enterprises (ME) (40.0%), and it was identified that they undertake in the area of Aesthetic Nursing (46.7%), Integrative and Complementary Practices (20.0%), Obstetric Nursing (20.0%), Stomatherapy (6.7%) and Commercialization of Health Products (6.7%). With regard to the length of time they had been working for the company, four (26.7%) had been working for one year, four (26.7%) had been working for between two and five years and seven (46.7%) had been working for more than six years. The majority (60.0%) have double employment, with the aim of adding extra income.

The type of staff was characterized by six (40.0%) entrepreneurs who had one employee, four (26.7%) had two to three, four (26.7%) had five and one (6.7%) had more than five employees. The predominant weekly working hours were 10 to 20 hours (53.3%).

Regarding economic characteristics, six had an initial investment in reais of between 11,000 and 50,000 (40.0%), five between 5,000 and 10,000 (33.3%), three more than 100,000 (30.0%) and one between 51,000 and 100,000 (6.7%). With regard to the income of the business in Brazilian minimum wages, six participants (40%) had a monthly income of more than five, three (20.0%) four to five, three (20.0%) two to four, two (13.3%) one to two and one (6.7%) less than one.

### Thematic Categories

**Figure 2:**
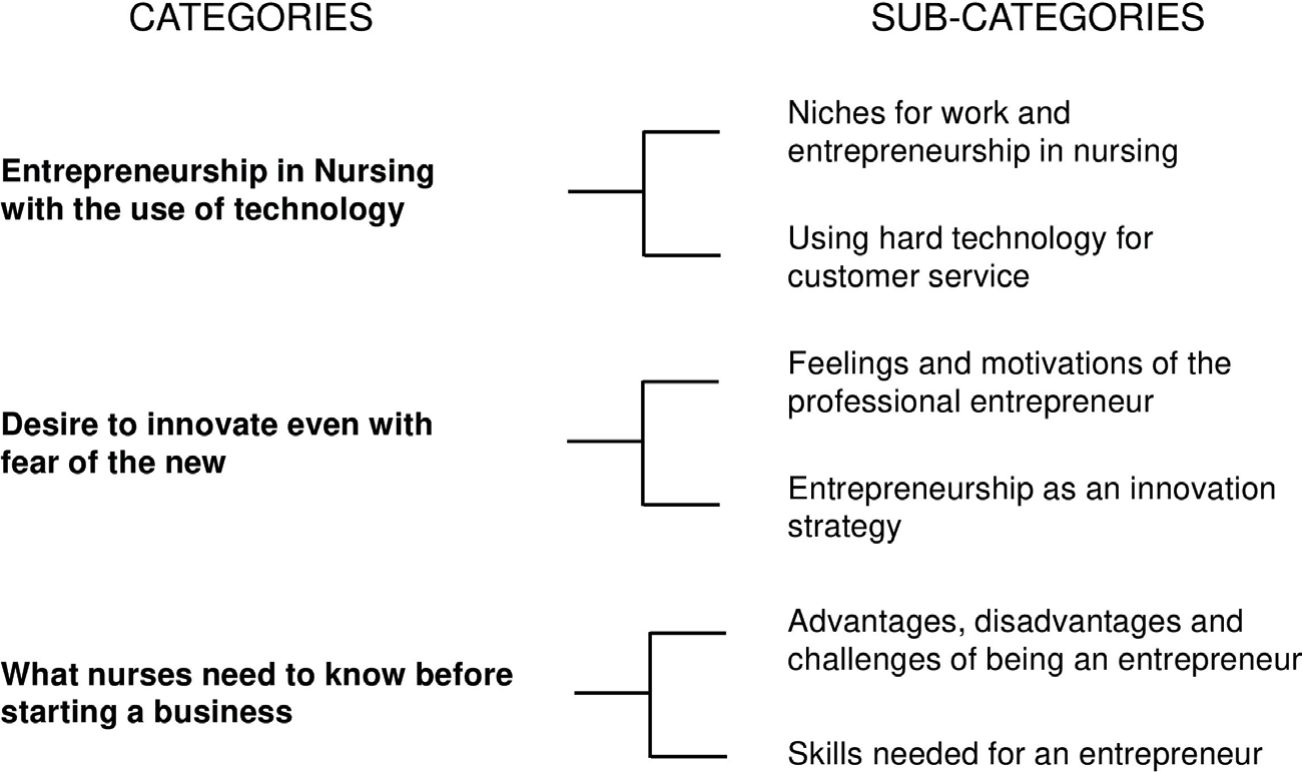
Thematic categories based on the content analysis proposed by Bardin.

Based on the analysis of the interviewees’ speeches, it was possible to identify three thematic categories, as shown in Figure 2.

### Entrepreneurship in Nursing with the use of Technology

This category gave rise to two subcategories: “Niches for working and entrepreneurship in nursing” and “Use of hard technology for customer service”.

#### Niches for Working and Entrepreneurship in Nursing

This subcategory revealed the main areas and/or services for nurses to work in, the reasons for seeking care and the approach to client care.

*I monitor low-risk and normal-risk prenatal care, nursing care during childbirth, postpartum and puerperium (E010).*

*There are areas of nursing that are facilitated by entrepreneurship. I work in dermatology, stomatherapy, podiatry and gerontology. (E015)*

*I provide health care related to integrative and complementary practices, such as acupuncture and suction cup therapy. (E01)*

*When patients come to me, they are very anxious about the results and it’s usually the service of last choice, so I explain the stages of treatment in detail [...]. (E01)*

*People usually come to me or contact me by cell phone because they’ve seen a post I’ve put on social media and they’re interested. (E05)*

*First of all, I carry out an initial assessment to generally address all the patient’s problems, then I determine the main needs that are hindering their well-being and then determine the best treatment and its benefits. (E01)*

*The way you treat your patient is something that sets your work apart from other professionals, so a humanized and polite treatment, a procedure carried out with excellence already makes your work stand out. (E012)*

#### Using Hard Technology for Customer Service

In this second subcategory, the participants described the technologies and/or procedures used, the benefits of using hard technologies and the difficulties in acquiring new technologies.

*In my office, I use a computer, speakers, electronic medical records, acupuncture devices: microstimulation or electrostimulation. (E01)*

*I work with natural gynecology, phytotherapy, phytoenergetics, crystal therapy, bioenergetics, psychodrama and some knowledge of shamanism. (E07)*

*I work with procedures focused on aesthetics, for example: skinbooster, botulinum toxin, jowl liposuction, intradermotherapy, PDO threads and body equipment too. (E014)*

*The use of these technologies will provide a shorter procedure time, but also produce comfort and satisfaction for my client. (E02)*

*The benefit lies in the high effectiveness of the results, where the procedures are safer and we have immediate and lasting results. (E07)*

*Lack of study on certain technology, how to handle and apply it at work, because this requires in-depth training. (E011)*

*The difficulty lies in adapting to new technologies, you always need to be up to date or even keep up with their advances. (E015)*

*Lack of capital is the biggest difficulty, how are we going to implement a new technology if we don’t have enough money? (E013)*

### Desire to Innovate Even with Fear of the New

From the second thematic category, two subcategories were constructed: “Feelings and motivations of the professional entrepreneur” and “Entrepreneurship as an innovation strategy”.

#### Feelings and Motivations of the Professional Entrepreneur

The first subcategory encompassed the feelings inherent to nurses during the process of entrepreneurship and the professional motivations for undertaking in nursing.

*I wish I had a visor so I couldn’t see so many possibilities for entrepreneurship in nursing, so many opportunities to solve health problems, it generates a certain amount of anguish. (E01)*

*First comes that feeling of fear because we don’t know if it’s going to work out. (E09)*

*I’ve always had a lot of resilience, today I’m a fearless person, I work in a legalized way and supported by the legislation of our class. (E012)*

*I’ve never been satisfied with nurses’ salaries, which is why I’ve tried to undertake to change this reality and show our profession that it is possible. (E02)*

*Ever since I was a child and even at university, I liked to sell candies, natural sandwiches, to get my own things and after I left university, I wanted to have my own business [...]. (E07)*

*It was necessary to take a risk, I began to see in hospitals the need to change this field of activity in order to promote differentiated care, so I looked for an area that I would enjoy working in. (E010)*

#### Entrepreneurship as an Innovation Strategy

In this subcategory, the professional’s willingness to innovate and the means of innovation used to make a difference at work were pointed out.

*You have to do it well [...], invest in marketing to help publicize your work and seek out scientific knowledge through courses or specializations in the area [...]. (E05)*

*So, it’s a question of knowing how to change marketing and work with the product you have, or even studying what and where you’re going to invest. And never miss opportunities [...]. (E012)*

*I monitor my patients through a messaging app or by phone call, where I ask if they are feeling better, schedule new appointments [...]. (E03)*

*I post my work on social media to show the procedures I carry out, if I put it in the window to attract customers’ attention, it’s interesting to do promotions too. (E010)*

*The best form of advertising is word of mouth marketing, where you value your work and are also referred by colleagues or friends. You have to bring new things to stimulate the search for service. (E014)*

### What Nurses Need to Know Before Starting a Business

This third category resulted in the subcategories: “Advantages, disadvantages and challenges of being an entrepreneur” and “Skills needed for an entrepreneur”.

#### Advantages, Disadvantages and Challenges of Being an Entrepreneur

The first subcategory comprises the advantages and disadvantages of being self-employed, as well as the challenges of becoming an entrepreneurial nurse.

*Choosing which activities I’m going to carry out, having greater control to act and seeking knowledge to qualify myself. (E07)*

*About being able to take your own actions without depending on anyone [...]. (E011)*

*A big advantage is the workload, because you set up your schedule at the times that work best for you. (E013)*

*A disadvantage is the excessive responsibility that falls on the professional when they work alone, so they need to involve other people to leverage the company’s development, with a lot of organization and planning. (E01)*

*The expense is huge, you have to be on your toes, so you don’t lose control and sink [...]. (E010)*

*When the number of appointments at the practice drops, so does my salary [...], which is why you never have a fixed income or don’t know how much you’re going to earn next month. (E014)*

*To remain a respected and reputable professional in a very persecuted profession. (E06)*

*Society doesn’t see nurses as qualified, skilled professionals who have also completed higher education and specialization courses, in order to be paid on a par with other health professions. (E07)*

*We don’t know how to charge for our services, for example, I remember that it took me a long time to be able to give my price and charge that amount for my work. (E09)*

*The training itself doesn’t encourage us to exercise autonomy in relation to nursing practices. Entrepreneurship isn’t covered in the degree course, which is why it requires professionals to seek training in order to be qualified. (E010)*

*The invisibility of the professional, in which people on the outside, the patient or client, are unaware of the roles that nurses play. (E015)*

#### Skills Needed for an Entrepreneur

The speeches highlighted the main skills needed to be a successful nurse entrepreneur and the need to continually seek knowledge beyond technical-scientific knowledge.

*Entrepreneurs have to be encouraged every day to leave their comfort zone, to always seek more, to be creative and persistent. (E01)*

*Being a self-taught professional, having a vision outside the box, mastering new experiences, giving things time to happen and being a leader. (E02)*

*You need to like to study, be dedicated to new practices, broaden your vision of care and be open to new technologies. (E08)*

*You need to be empowered, a leader rather than a boss, hard-working, up-to-date and innovative. (E014)*

*We have to keep investing in research because the foundation that strengthens us is really working with scientific evidence, but having the freedom to discuss experiences. (E08)*

*You need to have a postgraduate degree to enable you to do your job, where you will know how to carry out the procedure and provide the client with safety. (E013)*

## DISCUSSION

Entrepreneurship offers nurses the chance to increase their autonomy at work by applying innovative approaches, which require the use of creativity to develop new products or new ways of using existing products and/or services, which enables the transformation of care^(10)^. This idea corroborates the results of the present study, insofar as the nurses recruited sought technical-scientific knowledge beyond graduation in order to gain experiences related to improving new skills for successful entrepreneurship.

The first category “**Entrepreneurship in Nursing with the use of technologies**” shows the different areas of activity for nurse entrepreneurs and the applicability of technologies in the service to enable customer satisfaction. It is considered that this professional can work in different niches in the job market, such as offices caring for patients with wounds, stomatherapy, home care, aesthetics, private care in obstetrics and puerperium services. In addition, with constant technological advances, other niches have become available, such as offering courses and consultancies mediated by social networks^(10)^.

Unusual aspects of the care approach stand out, as mentioned by participant E07, who mentions the use of therapeutic resources based on traditional knowledge, such as natural gynecology and herbal medicine, to intervene in the prevention and treatment of various diseases. In addition, the use of complementary integrative practices was mentioned as an additional method to be applied by nurses with a focus on holistic and integral nursing care that can improve patient rehabilitation^(11)^. The legal support for working in these areas is ensured by the specialty registration, according to COFEN resolution no. 625/2020^(12)^. On the other hand, it requires nurses to be aware of the best available evidence and studies before incorporating these resources into their daily lives, in order to give scientific evidence to their practice.

In relation to the activity of nurses in the aesthetic area, presented in the speech of participant E014, COFEN resolution no. 626/2020^(13)^ regulates the performance of this professional as long as they are qualified in a postgraduate course, and can perform the following procedures: carboxytherapy, application of cosmetics and cosmeceuticals, dermopigmentation, lymphatic drainage, electrotherapy or electrothermophototherapy, combined ultrasound therapy, micropigmentation, cavitational ultrasound and vacuotherapy. Thus, the application of botulinum toxin, hyaluronic acid or biostimulators (cosmeceuticals) can be legally practiced by nurses in accordance with the aforementioned resolution, since it is an invasive technique that does not affect internal organs. It is also worth noting that the authorization for aesthetic nurses to carry out these invasive procedures differentiates them from other professionals in the aesthetics field, such as beauticians, thus corroborating a wider field of action for nursing.

Entrepreneurship in nursing is the search for new professional fronts that result in improved care, education, business or any other scenario in which nurses work^(14)^. In light of this statement, it is important to emphasize that nursing care must have a holistic vision to encompass all the problems that impair well-being, aiming to establish theoretical bases for clinical judgment of needs and care planning until health recovery. To do this, the nurse entrepreneur must make use not only of the theoretical framework that underpins the work of nurses, i.e. the Nursing Process, but also of innovations, such as the use of technology^(14)^.

These testimonials show that the use of technology in care tends to facilitate work, and this can contribute to other positive effects, such as increased efficiency, time savings, improved quality of care and standardization of procedures. While new technologies present themselves as a possibility to reduce the physical and mental burden on professionals in carrying out their activities^(15)^, the difficulty in keeping up to date and sometimes investing high amounts in acquiring these devices arise, which are obstacles in nurses’ daily lives^(11)^.

The second category “**Desire to innovate even with fear of the new**” portrays nurses’ feelings during their entrepreneurial career, the reasons that led them to undertake and the innovation strategies to show the difference in their work. Addressing this aspect, the literature^(16)^ states that many nurses at the start of their entrepreneurial careers do not consider themselves suitable for the role of entrepreneur due to gaps in their knowledge and skills in business during their academic training. During the transition as an entrepreneur, nurses experience some of the feelings inherent in the process, such as fear, insecurity, anxiety, stress and frustration. These emotions lead to learning how to mature and deal with situations. Linked to financial planning and business vision, a relevant achievement, in line with COFEN resolution 673/2021^(17)^, was the establishment of a table of minimum fees based on the Nursing Work Reference Unit (URTE in the Portuguese acronym). This table helps nurse entrepreneurs with the knowledge required for the services provided.

There are several motivational aspects for setting up a business: the opportunity comes from identifying a favorable opportunity for entrepreneurship and the need from the lack of options, from dissatisfaction in the market^(18)^. Given this, it is possible to make a comparison with the results of this study, which revealed that the nurses were driven by financial need and a desire for change in order to honor their place in the profession, with the possibilities of an attractive and daring venture.

At the beginning of the innovation process, nurses must rely on their ability to recognize a problem, create a solution and realize their potential, but also engage with marketing skills, business vision and establish a support team to facilitate the creation and implementation of ideas^(19)^. This statement allows us to understand that the creation of ideas is linked to the ability to identify problems and solve them, and shows how willing the professional is to innovate and seek knowledge.

From an innovation point of view, leadership in some health organizations gives nurses more opportunities for creative thinking, from which new ideas are generated, but risks in implementing them must be considered, which moves towards systematic changes and the development of innovation aimed at benefiting society^(20)^. This information discusses the importance of leadership in creating strategies that seek to improve care, which are unique and particular to arousing the interest of the population.

Lastly, the third category, “**What nurses need to know before starting a business**”, presents the advantages and disadvantages of being self-employed in the market, the challenges of entrepreneurship for nursing and the skills needed to be a successful professional in the management and practice of care. It can be highlighted that the benefits of an entrepreneurial career include the possibility of making a profit from your own business, along with management independence, as well as allowing greater flexibility in working hours and autonomy in decision-making. On the other hand, the disadvantages include competitiveness in the job market, a high level of responsibility and investment^(21)^.

Based on the negative effects of this business field, the entrepreneur must ensure high-performance management aiming to constant progress and advancement in their work, combined with financial planning to control spending. The need for financial control can also be seen in the fact that most of them have other jobs and sometimes have low financial returns from their ventures. This reinforces the perception of participant E07, who recalls the challenge that nurses still face in overcoming the medical-­centered model of care that exists in the social imagination.

The instability of the professional market impulses nurses to redesign their careers by starting their own business, but this choice can be hindered by several limiting factors, such as social and cultural prejudice against nurses having an office and/or clinic, the lack of a theoretical approach to this subject in universities and bureaucratic issues related to the administration and operation of the company^(21)^. From this standpoint, it can be seen that nurses are invisible due to the population’s lack of knowledge in relation to the professional’s qualification to carry out this practice, and also that this subject is incipient in the training of nurses, which requires a continuous search for training and qualification in order to master their skills.

It is necessary for nurses to value themselves and to know the importance of their profession so that they can convince clients of the relevance of their business, through essential attitudes, but above all through self-confidence, responsibility and a lot of dedication^(22)^. In this context, it should be emphasized that the entrepreneurial profile requires the necessary skills to become a successful professional, which is why it is important to have initiative, autonomy, decision-making, courage, resilience, empowerment, creativity, and above all, to adopt an innovative vision that goes beyond traditional standards, which are in line with the National Curriculum Guidelines (DCN in the Portuguese acronym)^(23)^.

Although the competencies set out in the DCNs are indispensable for an entrepreneur, the subject is still poorly covered in professional training, leaving gaps in the incentive to work in the area. Nursing schools should provide opportunities for this discussion in order to contribute to a professional who is better prepared for the needs of the job market. For example, a study carried out at a university in Istanbul concluded that students who showed a tendency towards entrepreneurship were more adaptable in their careers^(24)^.

Thus, the deficit in business knowledge is a challenge among entrepreneurial nurses who rarely have access to entrepreneurship courses and networks, which is why education is a pillar for successful business practice^(25,26)^. In this situation, it is necessary to bear in mind that progress comes from the continuous search for technical-scientific knowledge, in which the professional has the time, effort and dedication to keep up to date, because, in addition to the rapid technological changes that are currently provided, accessibility, through the communication channels present in everyone’s life, can be an ally for learning when used as a business management tool.

### Limitations of the Study

The data collection technique allowed interviews to be conducted through a network, but it does not represent all the entrepreneurial initiatives of nurses in Brazil. In this sense, a census could present a Brazilian panorama in a diagnostic way. It was observed that some nurses have greater capital power than others, either because of the size of their company or because of the monthly remuneration related to working hours. In line with this finding, a limitation of the study is the particular characteristics of each participant, such as access to entrepreneurship and the planning horizon of each of these nurses, which are an influencing factor in the process of building and consolidating a career.

## CONCLUSION

The experiences of the nurse entrepreneurs show that there are challenges to entrepreneurship in the nursing work process, which are obstacles to the recognition and appreciation of the professional, mainly the prejudice of society in relation to the technical-scientific competence to act as an entrepreneur, and the fact that the transversality of entrepreneurship has gaps in graduation. In addition, there are perceived financial difficulties in accessing technologies and implementing them in their work, while at the same time they value innovation strategies as an impetus for business success. Health technologies contribute to generating quality and safety in the services provided, optimizing processes and guaranteeing user satisfaction.

This study has the capacity to make a scientific contribution by revealing that encouraging nurses to become autonomous entrepreneurs is key for the profession because it enables them to conquer new fields of activity. Therefore, entrepreneurial practice can be a way of differentiating the Nursing category in the social imaginary by valuing autonomy, increasing the chances of innovating, creating and networking. This awakens the need for nurses to improve their skills in order to demystify how to deal with entrepreneurial practices.
